# Residential Ambient Traffic in Relation to Childhood Pneumonia among Urban Children in Shandong, China: A Cross-Sectional Study

**DOI:** 10.3390/ijerph15061076

**Published:** 2018-05-25

**Authors:** Jing Chang, Wei Liu, Chen Huang

**Affiliations:** 1 School of Environment and Architecture, University of Shanghai for Science and Technology, Shanghai 200093, China; changjingcj2004@163.com; 2 Department of Thermal Energy and Power Engineering, Shandong Jiaotong University, Jinan 250357, China; 3 Department of Building Science, Tsinghua University, Beijing 100084, China; lw1987@tsinghua.edu.cn; 4 Beijing Key Laboratory of Indoor Air Quality Evaluation and Control, Tsinghua University, Beijing 100084, China

**Keywords:** traffic, residence, pneumonia, China, children

## Abstract

Pneumonia is a leading cause of childhood death. Few studies have investigated associations between residential ambient environmental exposures and pneumonia. In January–April 2015, we conducted a cross-sectional study in Shandong Province (China) and collected 9597 (response rate: 78.7%) parent-reported questionnaires for 3–6-year-old children from 69 urban kindergartens. We then selected 5640 children who had never changed residence since birth and examined associations between residential ambient traffic-related facilities and childhood pneumonia considering residential characteristics. Prevalence of doctor-diagnosed pneumonia during lifetime-ever was 25.9%. In the multivariate logistic regression analyses, residence close to a main traffic road (adjusted odds ratio, 95% confidence interval: 1.23, 1.08–1.40) and automobile 4S shop (1.76, 1.16–2.67) within 200 m, residence close to a filling station within 100 m (1.71, 1.10–2.65; reference: >200 m), as well as having a ground car park in the residential community (1.24, 1.08–1.42) were significantly associated with childhood pneumonia. The cumulative numbers of these traffic-related facilities had a positive dose-response relationship with the increased odds of childhood pneumonia. These associations and dose-response relationships were stronger among boys and among children with worse bedroom ventilation status during the night. Associations of residence close to the main traffic road and ground car parks in the residential community with childhood pneumonia were stronger among children living in the 1st–3rd floors than those living on higher floors. Similar results were found in the two-level (kindergarten-child) logistic regression analyses. Our findings indicate that living near traffic-related facilities is likely a risk factor for childhood pneumonia among urban children. The child’s sex, bedroom floor level, and bedroom ventilation could modify associations of ambient traffic-related facilities with childhood pneumonia.

## 1. Background

Pneumonia is a leading cause of childhood morbidity and mortality [1,2]. In 2015, pneumonia caused about four million deaths and 35 million disability-adjusted life-years in children younger than five years of age [1,3].

A series of studies have reported that ambient traffic-related air pollution, which are mainly indicated by nitrogen oxides (NO_x_) and particulate matter with an aerodynamic diameter ≤10 μm (PM_10_), are associated with childhood pneumonia [4,5,6,7,8,9,10]. Several studies also have used roadways near residence or living near major roads as a proxy for ambient traffic-related pollution and investigated its association with childhood respiratory health [11,12,13,14,15,16,17,18,19,20,21]. Specifically, the birth-cohort studies within the European Study of Cohorts for Air Pollution Effects (ESCAPE) project found that doctor-diagnosed pneumonia in early childhood was significantly associated with exposure to ambient NO_2_ and PM_10_ [4,7]. A prospective cohort study for 3677 children from 12 Southern California communities found that compared to children living >1500 m from a freeway, those children living within 500 m of a freeway had notable deficits in 8-year growth of forced expiratory volume in 1 s (FEV1) and maximum mid-expiratory flow rate (MMEF) [16]. A systematic review reported significant associations of ambient particulate matter with an aerodynamic diameter ≤2.5 μm (PM_2.5_) exposure with acute lower respiratory infections in childhood [8]. Another systematic review also suggested a positive association of daily level of ambient air pollutants with childhood hospitalizations due to pneumonia [9].

However, most studies included in these systematic reviews were from high-income countries where concentrations of ambient air pollutants were relatively low, and similar studies from low-income and middle-income countries/regions where ambient air pollution is heavy are lacking [8,9], although, in China, ambient air pollution is a hot topic, and studies on associations between ambient air pollution and childhood respiratory diseases have rapidly increased in recent years [22,23,24,25,26,27,28,29,30,31]. Most of these studies reported positive associations of exposure to higher levels of ambient air pollutants with increased odds of pneumonia in childhood. Besides, most of these studies on associations of air pollution with childhood pneumonia did not consider the potential confounding from the building characteristics of residences and household environment factors, such as household ventilation [32,33], household dampness-related exposures [34,35,36], and environmental tobacco smoke [36,37], which have been reported to be significantly associated with childhood respiratory diseases.

In this paper, using parent-reported data for different indicators of residential ambient traffic and children’s health information from a cross-sectional study in Shandong province, China, we investigated associations of residential ambient traffic with doctor-diagnosed pneumonia in childhood among urban children. We hypothesized that there are significant associations between different indicators of residential ambient traffic and the increased odds of childhood pneumonia. Since several studies have reported sex-differences in associations of ambient air pollution and childhood health [38,39,40], we further separately conducted subanalyses of the target associations among boys and among girls. Besides, in our previous study, we found that bedroom floor level and bedroom ventilation could modify associations of residential ambient traffic with childhood respiratory health [41]. A recent study found that the diameter of inhalable particles in indoor air had decreasing trend as the floor level of residences increased [42]. We also conducted subanalyses of the target associations among children with different situations of bedroom floor level and bedroom ventilation.

## 2. Methods

### 2.1. Studied Population and Questionnaire

**Figure 1:**
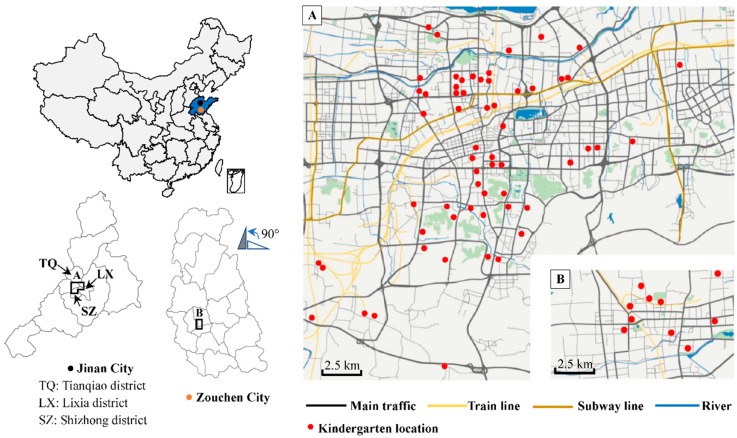
Locations of the surveyed kindergartens. (A): Studied area of Jinan city; (B): Studied area of Zouchen city.

During January–April 2015, basing on the China Children Homes Health (CCHH) study in ten large cities of China [43], we conducted a cross-sectional study in 69 urban kindergartens from Jinan city and Zoucheng city in Shandong province of China. In Jinan city, we surveyed all kindergartens in the Tianqiao district (*n* = 26) and in the Shizhong district (*n* = 28), and randomly surveyed five kindergartens in the Lixia district. In Zoucheng city, ten kindergartens in the urban district were randomly surveyed. Figure 1 shows locations and distributions of the surveyed kindergartens. The children normally attended the kindergartens closest to their residence. The children’s teachers distributed a standard questionnaire along with explanatory guidance to all children in the kindergartens (one family, one questionnaire) and returned them to us after the children’s parents filled out these questionnaires in the following days. A total of 12,202 parents of children were surveyed in these kindergartens and 9597 questionnaires (response rate: 78.7%) for children aged 3–6-year-old were finally collected. In the present study, we selected 5640 children who had never changed their residence since birth to examine the target associations.

In our questionnaire, questions about the child’s health history were translated from those in the International Study of Asthma and Allergies in Childhood (ISAAC) [44]. Questions about building characteristics of the residence, residential ambient environment, and family lifestyle behaviors were translated and modified from those in the Dampness in Building and Health (DBH) study in Sweden [45]. The full CCHH questionnaire was presented as supplemental material in a previous article [43] and in the present study. The readability and validity of the questionnaire has been verified by a pilot study in Chongqing in April 2010 [43].

Ethics approval and consent to participate: The ethical committee in the School of Public Health, Fudan University approved the questionnaire and research proposal (International Registered Number: IRB00002408 & FWA00002399). We informed participants of the purposes, details, and potential concerns of this study by written explanation. All participants consented in writing for themselves and their children, and voluntarily responded to the survey.

### 2.2. Assessment of Residential Traffic

In our questionnaire, basing on the question (*Is your residence within 200 m of a main traffic road/highway (yes vs. no)*) in the original CCHH questionnaire [43], we expanded 11 questions for different indicators with respect to residential ambient traffic-related exposures:

1. q1. Whether the residence close to main traffic road (yes vs. no)?
2. q1a. If q1 is yes, how far linear distance (m) between residence and main traffic road?
3. q1b. If q1 is yes, how many lane counts of the main traffic road near residence?
4. q1c. If q1 is yes, whether heavy truck passes through the main traffic road near residence?
5. q1d. If q1 is yes, whether bedroom faces to the main road near residence?
6. q2. Whether the residence close to filling station (yes vs. no)?
7. q2a. If q2 is yes, how far linear distance (m) between residence and filling station?
8. q3. Whether the residence close to automobile 4S shop (yes vs. no)?
9. q3a. If q3 is yes, how far linear distance (m) between residence and automobile 4S shop?
10. q4. Whether ground car park exists in the residential community (yes vs. no)?
11. q4a. If q4 is yes, how many cars are parked in the ground car park per day averagely?

Herein, automobile 4S shop was defined as a shop for the sale, spare parts, service, and survey of automobiles. The main road was defined as the roads for motor vehicle to connecting the major industrial and mining enterprises, major transportation hubs, and public places. If the response for q1, q2, or q3 was reported “yes” as well as the linear distance between residence and the traffic indicator was less than 200 m, we classified the children as having the corresponding traffic-related exposure, as reported in a previous study conducted in Shanghai, China [41]. If q4 was reported “yes”, we considered the child’s residence close to a ground car park. We also accumulated the number (*n*) of traffic indicators (including main traffic road, filling station, automobile 4S shop, and ground car park) near the residence for the analysis of a dose-response relationship between residence traffic exposure and the odd of childhood pneumonia. If none of these indicators was reported, *n* = 0; If any one of these indicators was reported, *n* = 1; and so on, thus *n* varied from zero to ≥3. Furthermore, for children whose residences were close to a main traffic road, filling station, and/or automobile 4S shop within 200 m, we stratified them into different subgroups according to the specific distances between the residence and these indicators and compared their risks of being diagnosed pneumonia during lifetime-ever. For children whose residential community had a ground car park, we also stratified them into different subgroups according to the number of parked cars and compared their pneumonia risks.

### 2.3. Assessment of Childhood Pneumonia

The childhood pneumonia we studied was diagnosed by a doctor and was reported by the children’s parents via the question (*Has your child ever been diagnosed with pneumonia by a doctor? (yes vs. no)*). Children were classified as having pneumonia during lifetime-ever when this question in their questionnaires was answered “yes”.

### 2.4. Covariates and Statistical Analyses

According to previous studies [18,41,45,46] and information from our questionnaire, we considered the following factors, which have been suggested to be associated with childhood respiratory diseases, as the potential covariates in the present study: sex (boys vs. girls), age (3 vs. 4 vs. 5 vs. 6-year-olds), residence-located area (Tianqiao district vs. Shizhong district vs. Lixia district vs. Zouchen city), family history of atopy (yes vs. no), residence ownership (owner vs. renter), breastfeeding duration (≤6 vs. >6 months), household dampness-related exposures (yes vs. no), household environmental tobacco smoke (yes vs. no), and household renovation during early lifetime (yes vs. no). Herein, family history of atopy was defined as that at least one of the child’s family members (siblings, parents, and/or grandparents) have had at least one of the following illnesses: asthma, eczema, and allergic nose or eye problems. We considered ownership of the current residence as an indicator of higher family socioeconomic status. Household dampness-related exposures were defined as that at least one of the following indicators was reported in the current residence: visible mold spots, visible damp stains, damp clothing/bedding, water damage, window pane condensation, and moldy odor. Household environmental tobacco smoke was defined as at least one smoker among family members living in the current residence. Here we considered the child’s sex, bedroom floor level (1st–3rd vs. 4th–6th vs. ≥7th floors), and frequency of opening bedroom windows during night (often vs. not often) as the potential effect modifiers. We categorized the child’s bedroom floor level according to the “Code for Design of Civil Buildings” in China [47]: 1st–3rd floors were defined as low floor level; 4th–6th floors were defined as medium floor level; ≥7th floors were defined as medium-high floor level. Frequency of opening bedroom windows during night was used to indicate the bedroom ventilation status. In the questionnaire, we asked family habit (often vs. sometimes vs. never) of bedroom ventilation in different seasons (spring, summer, autumn, and winter). We found that the family ventilation habits in different seasons had substantial correlations (see Supplemental Table S1). Therefore, if parents reported that the child’s bedroom windows were often opened during night in at least one season, we considered that the frequency of opening bedroom windows during night was often, and considered that their children’s bedroom ventilation was stronger than those families who did not often open bedroom windows.

The statistical analyses were performed by SPSS version 20.0 (IBM Inc., Armonk, NY, USA) and STATA 11.0 (STATA Corp., College Station, TX, USA). The Pearson’s chi-square test was used to compare the differences in pneumonia prevalence among children with different residential ambient traffic situations. We performed bivariate and multivariate logistic regression analyses to investigate crude and adjusted associations between different indicators of residential ambient traffic with childhood pneumonia by SPSS, respectively. In the multivariate logistic regression analyses (adjusted model 1), childhood pneumonia was set as a dependent variable; one indicator of residential ambient traffic was set as an independent variable; and the factors we mentioned above were set as covariates. To reduce the clustering effects within kindergarten and to check the target associations in the multivariate logistic regression analyses, we also conducted two-level (kindergarten-child) logistic regression analyses (adjusted model 2) using STATA. We performed the same analyses for the dose-response relationships between residential ambient traffic and the odds of childhood pneumonia. For associations and dose-response relationships, the traffic-related facilities were analyzed as categorical variables, with considering no exposure (or children with the longest linear distance between traffic-related facilities and residence) as reference categories. The associations and dose-response relationships were indicated by odds ratio (OR) and adjusted odds ratio (AOR) with 95% confidence intervals (CI). Significance was set at *p*-value < 0.05.

To indicate modification effects of residential ambient traffic with sex, bedroom floor level, or bedroom ventilation on associations of residential ambient traffic with childhood pneumonia, we firstly investigated the possibility of multiplicative interaction effects of residential ambient traffic with the bedroom floor level and bedroom ventilation habit on odds of childhood pneumonia, by including each of these two factors independently and their combined terms (residential ambient traffic × sex, residential ambient traffic × bedroom floor level, or residential ambient traffic × *family ventilation habit) as independent variables in the logistic regression models. If the corresponding *p*-value for the multiplicative item was <0.05, we considered that the combined two factors have interaction effect on childhood pneumonia. Secondly, we analyzed dose-response relationships between the cumulative numbers of residential ambient traffic-related facilities and childhood pneumonia in various subgroups, which were stratified according to the child’s sex, bedroom floor level, or bedroom ventilation habit in the logistic regression models.

## 3. Results

Table 1 shows the demographic data, sample distributions for covariates and pneumonia prevalence of the surveyed children.

Sample sizes for boys and girls had little difference. The proportions for 4-year-olds and for 5-year-olds children were both about one-third. Children both from Tianqiao district and from Shizhong district of Jinan city accounted for about 30%. A total of 9.4% of the surveyed children had a family history of atopy. A total of 72.2% and 19.1% children were from families who owned their current residence and were breastfed ≤6 months, respectively. A total of 80.9%, 52.0%, and 31.8% children had household dampness-related exposures, ETS exposure, and household renovation during early lifetime, respectively. A total of 50.9% and 38.1% children lived on the 1st–3rd floors and 4th–6th floors of their residential buildings, respectively. Here 71.7% children were from families who often opened bedroom windows during the night. The lifetime-ever prevalence of doctor-diagnosed pneumonia was 25.9%. With respect to residential ambient traffic (Table 2), 40.6%, 4.1%, and 2.1% of the surveyed residences close to main traffic road, filling station, and automobile 4S shop within 200 m, respectively. A total of 13.5% and 27.2% residences were close a main traffic road with a linear distance ≤50 m and close to ≥6 lanes traffic road, respectively. A total of 50.0% and 47.7% residences were close the main traffic roads for heavy trucks and faced the main traffic road, respectively. A total of 1.9% and 1.3% of residences were close to a filling station and an automobile 4S shop at a linear distance ≤100 m, respectively. A total of 59.8% residences had ground car parks in the residential community and therein 16.4% of these residences had >100 parked cars in the ground car park per day. Residences close to main traffic road, filling station, and automobile 4S shop within 200 m, as well as ground car parks in the residential community had no substantial correlations (Pearson’s correlation coefficient: 0.046–0.252; more data are shown in the Supplemental Table S2).

Compared to those children without ambient traffic-related facilities, significantly higher prevalences of childhood pneumonia were found among children whose residences were close to a main traffic road and had automobile 4S shops within 200 m, as well as among children whose residential communities had ground car parks (Table 2). Among those children whose residences were close to the main traffic road within 200 m, no significant differences in pneumonia prevalence were found among children whose residences were close to the main traffic road whether heavy trucks used the main traffic road and whether the child’s bedroom faced the main traffic road, as well as among children whose residences were close to main traffic roads with different lanes. Pneumonia prevalence among children whose residences were close to a filling station within 100 m was significantly higher than among other children. Although the differences were not statistically significant, pneumonia prevalences among children whose residences were close to a filling station within 100 m and within 101–200 m were higher than among children whose residences were close to the filling station >200 m. We found significant differences among children whose residential communities parked different car numbers per day, and the larger the average car number, the higher the pneumonia prevalence.

In the bivariate, multivariate, and two-level logistic regression analyses (Table 3), residences close to a main traffic road within 200 m (reference: >200 m) and residential communities having ground car parks had significant associations with the increased odds of childhood pneumonia. Residences close to a main traffic road within 51–100 m (reference: >200 m), residences close to a filling station within ≤100 m (reference: >200 m), and with >100 cars parked on the ground car park of the residence per day (reference: ≤20 cars) also had significant associations with increased odds of childhood pneumonia. Among children whose residences were close to a main traffic road within 200 m, whether heavy trucks used the main traffic road and whether their bedroom faces a main traffic road had no significant associations with childhood pneumonia. Besides, in the bivariate and multivariate logistic regression analyses, residences close to an automobile 4S shop within 200 m and ≤100 m (reference: >200 m) had significant associations with the increased odds of childhood pneumonia. 

Besides, a multivariate logistic regression analysis including all four traffic-related indicators in one model showed that the increased ORs (95%CI) of childhood pneumonia were 1.18 (1.03–1.36) for residences close to a main traffic road within 200 m, 1.06 (0.76–1.47) for residences close to a filling station within 200 m, 1.50 (0.95–2.35) for residences close to an automobile 4S shop within 200 m, and 1.21 (1.05–1.40) for residential communities having ground car parks (Table S3).

Furthermore, in the subanalyses (Table 4), main traffic road near residence within 200 m and having ground car park in the residential community had significant associations with the increased odds of childhood pneumonia among boys, among children whose bedrooms were on the 1st–3rd floors, and among children whose bedroom windows were not opened often during the night in all logistic regression analyses. 

Automobile 4S shop near residence within 200 m had a significant association with the increased odds of childhood pneumonia among boys among children whose bedrooms were on the 4th–6th floors in all logistic regression analyses. Filling station near residence within 200 m had no significant associations with the increased odds of childhood pneumonia among children of different sex, among children whose bedrooms were on different floor levels, and among children whose bedroom windows were opened or not opened often during the night, in the multivariate and two-level logistic regression analyses. Besides, in the bivariate and multivariate logistic regression analyses, main traffic road near the residence within 200 m had significant interactions with the child’s sex and bedroom ventilation habits on childhood pneumonia; an automobile 4S shop near residence within 200 m had significant interactions with bedroom ventilation habits on childhood pneumonia. In all logistic regression analyses, an automobile 4S shop near residence within 200 m had significant interactions with the child’s sex and bedroom floor level on childhood pneumonia. Ground car parks in the residential community had significant interactions with the child’s sex and bedroom ventilation habits on childhood pneumonia.

**Figure 2:**
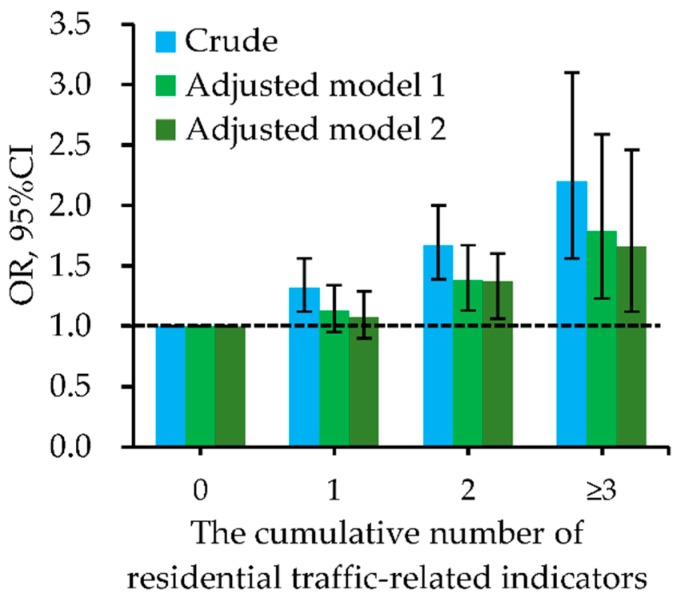
Dose-response relationships of the cumulative number of indictors for residential traffic within 200 m of the residence with odds of childhood pneumonia. Adjusted factors in model 1 (multivariate logistic regression analyses) included sex, age, residence-located area, family history of atopy, residence ownership, breastfeeding duration, household dampness-related exposures, household ETS, and household renovation during early lifetime. Adjusted factors in model 2 (two-level logistic regression analyses) included sex, age, family history of atopy, residence ownership, breastfeeding duration, household dampness-related exposures, household ETS, and household renovation during early lifetime. The supplemental Table S4 presented the detailed data for odds ratios (ORs) and their 95% confidence intervals (CIs).

**Figure 3:**
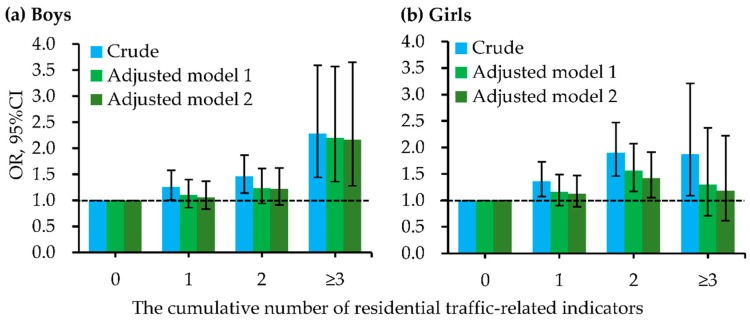
Dose-response relationships of the cumulative number of indictors for residential traffic within 200 m of the residence with odds of childhood pneumonia, stratified by the child’s sex. (**a**) Boys; (**b**) Girls. Adjusted factors in model 1 (multivariate logistic regression analyses) included age, residence-located area, family history of atopy, residence ownership, breastfeeding duration, household dampness-related exposures, household ETS, and household renovation during early lifetime. Adjusted factors in model 2 (two-level logistic regression analyses) included age, family history of atopy, residence ownership, breastfeeding duration, household dampness-related exposures, household ETS, and household renovation during early lifetime. The supplemental Table S5 presented the detailed data for odds ratios (ORs) and their 95% confidence intervals (CIs). The *p*-values for interaction in crude model, adjusted model 1, and adjusted model 2 were <0.001, 0.002, and 0.006, respectively.

**Figure 4:**
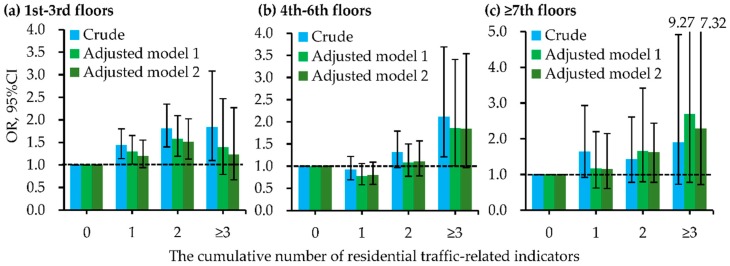
Dose-response relationships of the cumulative number of indictors for residential traffic within 200 m of the residence with odds of childhood pneumonia, stratified by bedroom floor level. (**a**) 1st-3rd floors; (**b**) 4th-6th floors; (**c**) ≥7th floors. Adjusted factors in model 1 (multivariate logistic regression analyses) included sex, age, residence-located area, family history of atopy, residence ownership, breastfeeding duration, household dampness-related exposures, household ETS, and household renovation during early lifetime. Adjusted factors in model 2 (two-level logistic regression analyses) included sex, age, family history of atopy, residence ownership, breastfeeding duration, household dampness-related exposures, household ETS, and household renovation during early lifetime. The supplemental Table S6 presented the detailed data for odds ratios (ORs) and their 95% confidence intervals (CIs). The *p*-values for interaction in crude model, adjusted model 1, and adjusted model 2 were 0.004, 0.082, and 0.108, respectively.

**Figure 5:**
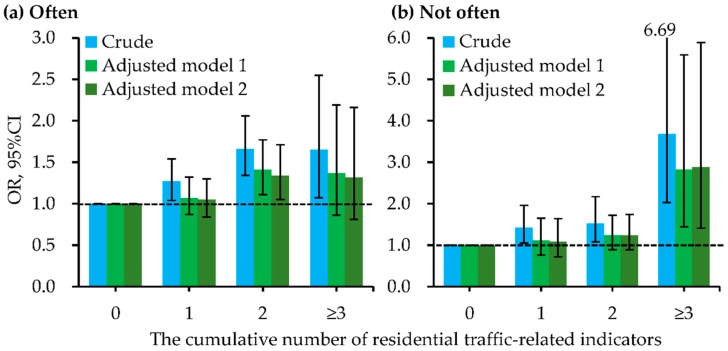
Dose-response relationships of the cumulative number of indictors for residential traffic within 200 m of the residence with odds of childhood pneumonia, stratified by family habit of bedroom ventilation (often or not often open the bedroom windows during night). (**a**) Often; (**b**) Not often. Adjusted factors in model 1 (multivariate logistic regression analyses) included sex, age, residence-located area, family history of atopy, residence ownership, breastfeeding duration, household dampness-related exposures, household ETS, and household renovation during early lifetime. Adjusted factors in model 2 (two-level logistic regression analyses) included sex, age, family history of atopy, residence ownership, breastfeeding duration, household dampness-related exposures, household ETS, and household renovation during early lifetime. The supplemental Table S7 presented the detailed data for odds ratios (ORs) and their 95% confidence intervals (CIs). The *p*-values for interaction in crude model, adjusted model 1, and adjusted model 2 were 0.001, 0.065, and 0.051, respectively.

Besides, the cumulative number of indictors for residential ambient traffic within 200 m of the residence had positive and significant dose-response relationships with the increased odds of childhood pneumonia (Figure 2 and Table S4). These dose-response relationships had significant differences between boys and girls (Figure 3 and Table S5). In all logistic regression analyses, the cumulative number of residential traffic-related indicators had significant interactions with the child’s sex on childhood pneumonia. Figure 4 and Table S6 present the dose-response relationships of the cumulative number of indicators for residential traffic with the odds of childhood pneumonia among children with different bedroom floor level. In the multivariate and two-level logistic regression analyses, the cumulative number of indicators for residential traffic and bedroom floor level had no significant interaction effects on childhood pneumonia. Associations of the cumulative number of indicators for residential traffic with childhood pneumonia were strongest among children living on the 4th–6th floors and having more than three traffic-related indicators within 200 m of the residence. In both multivariate and two-level logistic regression models (Figure 5 and Table S7), the cumulative number of indicators for residential traffic and bedroom ventilation habit had nearly significant interaction effects on childhood pneumonia (*p*-value < 0.1). For children who have more than three traffic-related indicators within 200 m of the residence, associations of the cumulative number of indicators for residential traffic with childhood pneumonia were notably stronger among children whose families did not often open than did often open the bedroom windows.

## 4. Discussion

In this cross-sectional study, we found that childhood pneumonia had positive and significant associations with residences close to main traffic roads or automobile 4S shops within 200 m, and residential communities having ground car parks. Residence close to a filling station within 100 m had a significant association with the increased odd of childhood pneumonia. The cumulative number of these indictors for residential ambient traffic had positive and significant dose-response relationships with the increased odds of childhood pneumonia. Associations of traffic-related indicators and the cumulative number of these indicators with childhood pneumonia were generally stronger among boys than girls, as well as among children whose families did not often open than those who often opened the bedroom windows. Associations of residence close to a main traffic road within 200 m and having a ground car park in the residential community with childhood pneumonia were stronger among children living on the 1st–3rd floors than among children living on the 4th–6th floors and ≥7th floors. Associations of automobile 4S shops near residences within 200 m with childhood pneumonia were stronger among children living on the 4th–6th floors and ≥7th floors than among children living on the 1st–3rd floors. The day-average numbers of car parked in the ground car park in the residential community also had dose-response relationships with the increased odds of childhood pneumonia. Among children whose residences were close to main traffic road ≤200 m, lane counts of the road, heavy truck passing along the road and bedroom facing the road had no significant associations with childhood pneumonia.

Our findings with regard to the significant associations of different traffic-related facilities near the residence and childhood pneumonia were partly consistent with several previous studies on associations between ambient air pollution and respiratory diseases/symptoms [4,6,7,8,9,26,27,28,41,48,49]. Specifically, the meta-analysis of 10 birth-cohort studies within the ESCAPE project found that parent-reported doctor-diagnosed pneumonia in early childhood had statistically significant associations with ambient annual-average NO_2_ and PM_10_ [7]. A systematic review reported significant associations of ambient PM_2.5_ exposure with acute lower respiratory infections in childhood [8]. Another systematic review also suggested ambient air pollutants were significantly associated with the increased odds of childhood hospitalization due to pneumonia [9]. A case-crossover study in Jinan city of China also found that pediatric hospitalization for pneumonia was significantly associated with the elevated PM_2.5_ concentrations in the day before hospitalization and the elevated PM_10_ concentrations in the two days before hospitalization [28]. A cross-sectional study for 3–6-year-olds children in Changsha city of China found that the elevated NO_2_ concentration during lifetime-ever had significant associations with the increased odds of childhood pneumonia [27]. A similar study in Shanghai, China also reported that living close to high traffic roads or highways within 200 m of the residence had positive association with the increased odd of childhood pneumonia [41]. These findings suggested that living near traffic-related facilities could be a risk factor for childhood pneumonia. 

A novel finding in the present study was that lane count of the main traffic road, heavy truck use on the road, and bedroom facing the road had no significant association with childhood pneumonia among children whose residences were close to a main traffic road ≤200 m. To our best knowledge, there was no previous study having the similar findings for childhood pneumonia with the present study. These findings also were inconsistent with several previous studies on associations of linear distances between residence and main traffic road with childhood asthmatic and allergic symptoms [13,50,51,52,53]. Specifically, the phase three of the ISAAC study found that high frequency of self-reported truck traffic on the residential street had positive associations and significant exposure-response relationships with the prevalence of symptoms for childhood asthma, rhinitis, and eczema [13]. The Cincinnati Childhood Allergy and Air Pollution Study (CCAAPS) found that infants living stop-and-go bus and truck traffic <100 m (reference: unexposed infants) was significantly increased odds of wheezing symptoms among infants <1 year-old [52]. This study also suggested that distance between residence and traffic as well as traffic type had stronger associations with wheezing in early infancy than traffic volume [52]. Since our studied outcome was doctor-diagnosed pneumonia during the child’s lifetime-ever, the above findings seemingly suggest that associations of long-term exposure to traffic-related facilities on childhood doctor-diagnosed pneumonia could be similar among children whose residences are close to main traffic roads of different types, whereas associations of traffic-related facilities on symptoms of these diseases could interact with the distances between residence and ambient traffic roads. More related studies are warranted.

Besides, we report some novel findings with respect to interaction effects. Consistent with most previous studies [38,39,40], we find notable differences in associations of residential ambient traffic with childhood pneumonia between boys and girls. These findings suggest that effects of residential ambient traffic-related facilities on childhood pneumonia could be stronger in boys than in girls in urban area of Shandong Province, China.

As a similar study in Shanghai, China [41], we found that the child’s bedroom floor level or bedroom ventilation status with the residential ambient traffic had significant or nearly significant interaction effects on childhood pneumonia. With respect to the bedroom floor level, several studies have reported that ambient/indoor air pollution could differ on different floors of residential buildings [42,54,55,56]. A simulation study for a high-rise building in Hong Kong suggested that the pathway of pollutant migration was dominated by wind-structure interaction [56]. Another study found that indoor levels of polycyclic aromatic hydrocarbons (PAH) and carbon black (CB, an indicator of ambient heavy traffic) from ambient air declined with the increased floor level [54]. These findings suggested that increased odds of childhood pneumonia due to ambient traffic-related facilities could depend on the residence floor level, that could be related with the natural ventilation status and thus be associated with the household air quality [54,55]. However, to our best knowledge, except for the study in Shanghai, China [41], there is no study yet considering the impact of the bedroom floor level on children’s health and its interaction effect with ambient traffic on children’s health. In the present study, the significantly increased odds of pneumonia due to residence close to a main road and due to having a ground car park in the residential community were consistently found among children living on 1st–3rd floors in different logistic regression analyses. Our findings seemingly suggested that traffic-related facilities had a greater impact on the increased odds of childhood pneumonia among children living on a low floor level than on a high floor of the residential building.

With respect to bedroom ventilation status, often opening bedroom windows is a common method to improve household ventilation. Increasing household natural ventilation could effectively reduce indoor air pollution [57]. This explanation supported our findings in the present study that associations of residential ambient traffic-related facilities t with childhood pneumonia were stronger among children whose families did not often open the bedroom windows than among children whose families often opened the bedroom windows during the night. Therefore, like the bedroom floor level, we suggest that family habits of opening bedroom windows also should be considered in related studies on associations of residential ambient traffic with childhood respiratory diseases. These findings also further support that residential ambient traffic-related facilities could be risk factors for childhood pneumonia.

Some limitations exist in the present study. First, all data we analyzed were collected from parent-reported questionnaires. Pneumonia was defined as a single positive answer to a question. These data we analyzed might have reporting error and bias. Second, we did not consider the environmental exposures in the kindergarten, where the children spend a notable fraction of their time, and these environmental exposures are also likely to affect the child’s respiratory health [57]. Third, the sample sizes for some subgroups we analyzed were small.

Nevertheless, this study has several strengths. First, it is one of the first large cross-sectional studies in China on the associations of residential ambient traffic with childhood pneumonia. The questionnaire we used has been used in several previous studies [22,23,43,45,58] and some studies have confirmed the validity of the questionnaire [59,60]. The primary questionnaire also has been tested in a pilot study of 100 children in April 2010 in Chongqing, China and thereafter its readability has been improved [43]. Second, the high response rate and large sample sizes assured that the analyzed data had high representation for the studied population and likely have little selection bias. The large number of participants also could dilute the self-reporting methodology bias for both pneumonia and environmental factors. Third, in addition to using main traffic road to indicate residential ambient traffic-related condition as many previous studies [12,50,51,52,53], we added three traffic-related facilities (filling station, automobile 4S shop, and ground car park) and obtained the detailed linear distance between residences and main traffic roads as well as type of the traffic road and ground car park in the residential community. Fourth, except for the similar study in Shanghai, China [41], this is the first study to analyze the interactions of the child’s sex, bedroom floor level, and family ventilation habits on the associations between residential ambient traffic-related exposures and childhood pneumonia. Fifth, we have considered several household environmental factors (such as household decoration and dampness-related exposure) as potential confounders in the analyses of the target associations.

## 5. Conclusions

Residential ambient traffic facilities are related with childhood pneumonia among urban children in Shandong, China. Residence close to main traffic road and automobile 4S shop within 200 m, residence close to filling station within 100 m, and having ground car park in the residential community are associated with the higher odds of childhood pneumonia. The child’s sex, bedroom floor level, and bedroom ventilation could affect associations between residential ambient traffic and childhood pneumonia. These associations could be stronger among boys, among children living on low floor levels, and among children with worse bedroom ventilation.

## Figures and Tables

**Table 1:** Demographic data, covariates, and pneumonia prevalence.

Items	Sample Size, *n* (%) ^a^
Total	5640 (100.0)
Sex	
Boys	2906 (52.3)
Girls	2650 (47.7)
Age	
3-year-olds	945 (16.9)
4-year-olds	1862 (33.2)
5-year-olds	1866 (33.3)
6-year-olds	931 (16.6)
Residence-located area	
Tianqiao district	1724 (30.6)
Shizhong district	1688 (29.9)
Lixia district	896 (15.9)
Zouchen city	1332 (23.6)
Family history of atopy	
Yes	524 (9.4)
No	5076 (90.6)
Residence ownership	
Owner	3971 (72.2)
Renter	1532 (27.8)
Breastfeeding duration	
≤6 months	1057 (19.1)
>6 months	4472 (80.9)
Household dampness-related exposure	
Yes	4515 (81.2)
No	1047 (18.8)
Household environmental tobacco smoke (ETS)	
Yes	2892 (52.0)
No	2670 (48.0)
Household renovation during early lifetime	
Yes	1703 (31.8)
No	3646 (68.2)
The child’s bedroom floor level	
1st–3rd floors	2723 (50.9)
4th–6th floors	2040 (38.1)
≥7th floors	586 (11.0)
Frequency of opening bedroom windows during night	
Often	3950 (71.7)
Not often	1559 (28.3)
Pneumonia prevalence	
Yes	1429 (25.9)
No	4092 (74.1)

^a^ Due to missing data, sample sums in some items could be smaller than the total number.

**Table 2:** Pneumonia prevalence among children with different situations of residential traffic.

Indictors for Residential Traffic	Sample Size, *n* (%)	Prevalence, *n* (%)	*p*-Value ^a^
Main traffic road near residence within 200 m			
Yes	2225 (40.6)	**630 (29.0)**	
No	3259 (59.4)	**762 (23.9)**	**<0.001**
Linear distance between residence and the main traffic road			
≤50 m	669 (13.5)	**186 (28.6)**	
51–100 m	475 (9.6)	**139 (29.6)**	
101–200 m	555 (11.2)	**149 (27.2)**	
>200 m	3259 (65.7)	**762 (23.9)**	**0.005**
Lane counts of the main traffic road near residence within 200 m			
≤2 lanes	499 (31.3)	123 (25.5)	
3–5 lanes	662 (41.5)	197 (30.2)	
≥6 lanes	434 (27.2)	134 (31.4)	0.109
Heavy truck passed through the main traffic road near residence within 200 m			
Yes	827 (50.0)	222 (27.3)	
No	828 (50.0)	235 (29.0)	0.445
Bedroom faced to the main traffic road near residence within 200 m			
Yes	802 (47.7)	221 (28.3)	
No	879 (52.3)	249 (28.7)	0.874
Filling station near residence within 200 m			
Yes	234 (4.1)	71 (30.7)	
No	5189 (95.7)	1302 (25.6)	0.081
Linear distance between residence and the filling station			
≤100 m	105 (1.9)	**41 (39.0)**	
101–200 m	129 (2.4)	**30 (23.8)**	
>200 m	5189 (95.7)	**1302 (25.6)**	**0.007**
Automobile 4S shop near residence within 200 m			
Yes	117 (2.1)	**41 (35.3)**	
No	5327 (97.9)	**1338 (25.6)**	**0.018**
Linear distance between residence and the automobile 4S shop			
≤100 m	72 (1.3)	26 (36.6)	
101–200 m	45 (0.8)	15 (33.3)	
>200 m	5327 (97.9)	1338 (25.6)	0.056
Ground car park in the residential community			
Yes	3234 (59.8)	**909 (28.6)**	
No	2175 (40.2)	**467 (21.9)**	**<0.001**
Average number of cars parked in the ground car park per day			
≤20	1014 (36.2)	**251 (25.2)**	
21–50	795 (28.4)	**233 (29.7)**	
51–100	532 (19.0)	**159 (30.2)**	
>100	459 (16.4)	**151 (34.0)**	**0.004**

^a^ In the Pearson’s chi-square test; Bold indicates significance (*p*-value < 0.05).

**Table 3:** Associations of childhood pneumonia with residential traffic in the logistic regression analyses.

Indictors for Residential Traffic	OR, 95%CI (*p*-Value) ^a^		
	Crude	Adjusted Model 1 ^b^	Adjusted Model 2 ^c^
Main traffic road near residence within 200 m			
No	1.00	1.00	1.00
Yes	**1.30, 1.15–1.47 (<0.001)**	**1.23, 1.08–1.40 (0.002)**	**1.21, 1.05–1.38 (0.007)**
Linear distance between residence and the main traffic road			
>200 m	1.00	1.00	1.00
101–200 m	1.20, 0.97–1.47 (0.089)	1.13, 0.91–1.41 (0.277)	1.09, 0.87–1.37 (0.452)
51–100 m	**1.34, 1.09–1.67 (0.007)**	**1.33, 1.06–1.68 (0.013)**	**1.30, 1.03–1.64 (0.030)**
≤50 m	**1.28, 1.06–1.55 (0.010)**	1.16, 0.94–1.42 (0.163)	1.10, 0.90–1.36 (0.355)
Lane counts of the main traffic road near residence within 200 m			
≤2 lanes	1.00	1.00	1.00
3–5 lanes	1.26, 0.97–1.64 (0.085)	1.24, 0.93–1.66 (0.144)	1.24, 0.92–1.68 (0.156)
≥6 lanes	1.34, 1.00–1.78 (0.050)	1.32, 0.96–1.82 (0.087)	**1.40, 1.01–1.94 (0.045)**
Heavy truck passed through the main traffic road near residence within 200 m			
No	1.00	1.00	1.00
Yes	0.92, 0.74–1.14 (0.445)	0.79, 0.63–1.01 (0.053)	0.83, 0.65–1.06 (0.135)
Bedroom faced to the main traffic road near residence within 200 m			
No	1.00	1.00	1.00
Yes	0.98, 0.79–1.22 (0.874)	1.02, 0.81–1.28 (0.863)	1.02, 0.81–1.29 (0.840)
Filling station near residence within 200 m			
No	1.00	1.00	1.00
Yes	1.29, 0.97–1.72 (0.081)	1.22, 0.89–1.66 (0.222)	1.17, 0.85–1.61 (0.338)
Linear distance between residence and the filling station			
>200 m	1.00	1.00	1.00
101–200 m	0.91, 0.60–1.38 (0.648)	0.89, 0.57–1.39 (0.615)	0.87, 0.56–1.38 (0.567)
≤100 m	**1.86, 1.25–2.77 (0.002)**	**1.71, 1.10–2.65 (0.017)**	**1.60, 1.02–2.52 (0.039)**
Automobile 4S shop near residence within 200 m			
No	1.00	1.00	1.00
Yes	**1.59, 1.08–2.33 (0.018)**	**1.76, 1.16–2.67 (0.008)**	1.43, 0.94–2.20 (0.098)
Linear distance between residence and the automobile 4S shop			
>200 m	1.00	1.00	1.00
101–200 m	1.45, 0.78–2.71 (0.238)	1.80, 0.92–3.50 (0.086)	1.52, 0.77–2.99 (0.223)
≤100 m	**1.68, 1.03–2.73 (0.035)**	**1.74, 1.03–2.95 (0.039)**	1.37, 0.80–2.35 (0.252)
Ground car park in the residential community			
No	1.00	1.00	1.00
Yes	**1.43, 1.26–1.62 (<0.001)**	**1.24, 1.08–1.42 (0.002)**	**1.20, 1.04–1.39 (0.014)**
Average number of car parked in the ground car park per day			
≤20	1.00	1.00	1.00
21–50	**1.26, 1.02–1.55 (0.034)**	1.21, 0.96–1.52 (0.100)	1.24, 0.98–1.56 (0.075)
51–100	**1.29, 1.02–1.63 (0.035)**	1.21, 0.94–1.56 (0.146)	1.25, 0.97–1.63 (0.088)
>100	**1.53, 1.20–1.95 (0.001)**	**1.41, 1.08–1.84 (0.011)**	**1.49, 1.13–1.95 (0.004)**

^a^ OR: odds ratio; CI: confidence interval; Bold indicates significance (*p*-value < 0.05). ^b^ Multivariate logistic regression analyses with adjustment for the child’s sex, age, residence-located area, family history of atopy, residence ownership, breastfeeding duration, household dampness-related exposures, household ETS, and household renovation during early lifetime. ^c^ Two-level (kindergarten-child) logistic regression analyses with adjustment for the child’s sex, age, family history of atopy, residence ownership, breastfeeding duration, household dampness-related exposures, household ETS, and household renovation during early lifetime.

**Table 4:** Associations of childhood pneumonia with residential traffic in the logistic regression analyses in the subgroups, stratified by the child’s sex, bedroom floor level, and bedroom ventilation habit.

Indictors for Residential Traffic	OR, 95%CI (*p*-Value) [*p-*Value for Interaction] ^a^		
	Crude	Adjusted Model 1 ^b^	Adjusted Model 2 ^c^
**1. Main traffic road near residence within 200 m (yes vs. no)**			
Stratified by the child’s sex			
Boys	**1.42, 1.19–1.71 (<0.001)**	**1.32, 1.09–1.60 (0.005)**	**1.27, 1.04–1.55 (0.018)**
Girls	1.18, 1.00–1.40 (0.055) **[0.009]**	1.15, 0.96–1.38 (0.124) **[0.035]**	1.14, 0.94–1.37 (0.176) [0.058]
Stratified by bedroom floor level			
1st–3rd floors	**1.37, 1.14–1.63 (0.001)**	**1.29, 1.06–1.56 (0.010)**	**1.27, 1.04–1.54 (0.019)**
4th–6th floors	**1.24, 1.01–1.52 (0.039)**	1.18, 0.95–1.47 (0.130)	1.15, 0.92–1.43 (0.228)
≥7th floors	1.14, 0.79–1.63 (0.494) **[0.015]**	1.09, 0.73–1.61 (0.681) [0.124]	1.08, 0.73–1.60 (0.683) [0.197]
Stratified by bedroom ventilation habit (open the bedroom windows during night)			
Often	**1.26, 1.01–1.59 (0.047)**	1.16, 0.90–1.49 (0.257)	1.22, 0.93–1.61 (0.142)
Not often	**1.29, 1.11–1.49 (0.001) [0.012]**	**1.23, 1.05–1.44 (0.011) [0.013]**	**1.21, 1.03–1.42 (0.022)** [0.083]
**2. Filling station near residence within 200 m (yes vs. no)**			
Stratified by the child’s sex			
Boys	1.41, 0.96–2.06 (0.078)	1.42, 0.95–2.14 (0.087)	1.37, 0.90–2.08 (0.137)
Girls	1.08, 0.69–1.70 (0.724) **[0.049]**	0.98, 0.60–1.61 (0.928) [0.082]	0.96, 0.58–1.60 (0.885) [0.093]
Stratified by bedroom floor level			
1st–3rd floors	1.17, 0.76–1.81 (0.472)	1.05, 0.65–1.69 (0.848)	0.93, 0.57–1.51 (0.758)
4th–6th floors	1.18, 0.74–1.87 (0.483)	1.23, 0.75–2.02 (0.422)	1.21, 0.72–2.02 (0.467)
≥7th floors	1.28, 0.59–2.81 (0.530) [0.243]	1.34, 0.56–3.17 (0.511) [0.309]	1.31, 0.56–3.10 (0.533) [0.241]
Stratified by bedroom ventilation habit (open the bedroom windows during night)			
Often	1.14, 0.80–1.63 (0.470)	1.12, 0.77–1.65 (0.553)	1.10, 0.74–1.63 (0.626)
Not often	**1.75, 1.07–2.87 (0.026) [0.002]**	1.42, 0.82–2.45 (0.209) [0.700]	1.33, 0.76–2.33 (0.310) [0.678]
**3. Automobile 4S shop near residence within 200 m (yes vs. no)**			
Stratified by the child’s sex			
Boys	**2.09, 1.27–3.44 (0.003)**	**2.55, 1.49–4.38 (0.001)**	**2.12, 1.22–3.69 (0.008)**
Girls	0.88, 0.45–1.73 (0.705) **[0.002]**	1.05, 0.52–2.12 (0.883) **[<0.001]**	0.87, 0.43–1.78 (0.702) **[0.005]**
Stratified by bedroom floor level			
1st–3rd floors	1.07, 0.59–1.95 (0.821)	0.95, 0.48–1.90 (0.884)	0.73, 0.36–1.49 (0.389)
4th–6th floors	**2.49, 1.36–4.58 (0.002)**	**2.77, 1.49–5.16 (0.001)**	**2.47, 1.31–4.68 (0.005)**
≥7th floors	2.04, 0.61–6.79 (0.235) **[0.002]**	3.62, 0.83–15.85 (0.088) **[<0.001]**	3.63, 0.83–15.87 (0.087) **[0.002]**
Stratified by bedroom ventilation habit (open the bedroom windows during night)			
Often	1.26, 0.75–2.10 (0.388)	1.47, 0.84–2.57 (0.178)	1.17, 0.66–2.08 (0.583)
Not often	**1.99, 1.08–3.69 (0.026) [0.008]**	1.81, 0.94–3.50 (0.076) **[0.016]**	1.60, 0.81–3.16 (0.172) [0.068]
**4. Ground car park in the residential community (yes vs. no)**			
Stratified by the child’s sex			
Boys	**1.46, 1.21–1.77 (<0.001)**	**1.26, 1.03–1.54 (0.022)**	**1.22, 1.01–1.49 (0.044)**
Girls	**1.39, 1.16–1.66 (<0.001) [<0.001]**	**1.22, 1.01–1.48 (0.040) [0.007]**	1.21, 0.98–1.49 (0.075) **[0.022]**
Stratified by bedroom floor level			
1st–3rd floors	**1.48, 1.23–1.77 (<0.001)**	**1.34, 1.10–1.63 (0.003)**	**1.25, 1.02–1.53 (0.035)**
4th–6th floors	1.22, 0.98–1.52 (0.075)	1.05, 0.83–1.33 (0.679)	1.12, 0.88–1.43 (0.344)
≥7th floors	1.29, 0.84–1.99 (0.250) **[0.013]**	1.09, 0.68–1.75 (0.718) [0.293]	1.08, 0.68–1.74 (0.735) [0.330]
Stratified by bedroom ventilation habit (open the bedroom windows during night)			
Often	**1.31, 1.12–1.52 (0.001)**	1.16, 0.98–1.37 (0.079)	1.14, 0.96–1.35 (0.127)
Not often	**1.71, 1.34–2.18 (<0.001) [0.017]**	**1.38, 1.06–1.80 (0.016) [0.042]**	**1.38, 1.06–1.81 (0.018) [0.035]**

^a^ OR: odds ratio; CI: confidence interval; Bold indicates significance (*p*-value < 0.05). ^b^ Multivariate logistic regression analyses with adjustment for the child’s sex (excluded when the subgroups were stratified by the child’s sex), age, residence-located area, family history of atopy, residence ownership, breastfeeding duration, household dampness-related exposures, household ETS, and household renovation during early lifetime. ^c^ Two-level (kindergarten-child) logistic regression analyses with adjustment for the child’s sex (excluded when the subgroups were stratified by the child’s sex), age, family history of atopy, residence ownership, breastfeeding duration, household dampness-related exposures, household ETS, and household renovation during early lifetime.

## Supplementary Materials

The following are available online at http://www.mdpi.com/1660-4601/15/6/1076/s1, Questionnaire 1: Questionnaire used in CCHH phase I, Table S1: Correlation among family habits of window opening in different seasons, Table S2: Correlation among residential ambient traffic-related indicators, Table S3: Associations of indictors for residential traffic with childhood pneumonia in the logistic analysis for multiple exposures, Table S4: Dose-response relationships of the cumulative number of indictors for residential traffic with odds of childhood pneumonia, Table S5: Dose-response relationships of the cumulative number of indictors for residential traffic with odds of childhood pneumonia, stratified by the child’s sex, Table S6: Dose-response relationships of the cumulative number of indictors for residential traffic with odds of childhood pneumonia, stratified by bedroom floor level, Table S7: Dose-response relationships of the cumulative number of indictors for residential traffic with odds of childhood pneumonia, stratified by family habit of bedroom ventilation.

## Availability of Data and Materials

The datasets during and/or analyzed during the current study available from the corresponding author on reasonable request.

## References

[1] GBD 2015 LRI Collaborators (2017). Estimates of the global, regional, and national morbidity, mortality, and aetiologies of lower respiratory tract infections in 195 countries: A systematic analysis for the Global Burden of Disease Study 2015. Lancet Infect. Dis..

[2] Walker C.L., Rudan I., Liu L., Nair H., Theodoratou E., Bhutta Z.A., O’Brien K.L., Campbell H., Black R.E. (2013). Global burden of childhood pneumonia and diarrhoea. Lancet.

[3] Brauer M., Freedman G., Frostad J., van Donkelaar A., Martin R.V., Dentener F., van Dingenen R., Estep K., Amini H., Apte J.S. (2016). Ambient air pollution exposure estimation for the Global Burden of Disease 2013. Environ. Sci. Technol..

[4] Aguilera I., Pedersen M., Garcia-Esteban R., Ballester F., Basterrechea M., Esplugues A., Fernández-Somoano A., Lertxundi A., Tardón A., Sunyer J. (2013). Early-life exposure to outdoor air pollution and respiratory health, ear infections, and eczema in infants from the INMA study. Environ. Health Perspect..

[5] Fuertes E., MacIntyre E., Agius R., Beelen R., Brunekreef B., Bucci S., Cesaroni G., Cirach M., Cyrys J., Forastiere F. (2004). Associations between particulate matter elements and early-life pneumonia in seven birth cohorts: Results from the ESCAPE and TRANSPHORM projects. Int. J. Hyg. Environ. Health.

[6] Lipfert F.W. (2017). A critical review of the ESCAPE project for estimating long-term health effects of air pollution. Environ. Int..

[7] MacIntyre E.A., Gehring U., Mölter A., Fuertes E., Klümper C., Krämer U., Quass U., Hoffmann B., Gascon M., Brunekreef B. (2014). Air pollution and respiratory infections during early childhood: An analysis of 10 European birth cohorts within the ESCAPE project. Environ. Health Perspect..

[8] Mehta S., Shin H., Burnett R., North T., Cohen A.J. (2013). Ambient particulate air pollution and acute lower respiratory infections: A systematic review and implications for estimating the global burden of disease. Air Qual. Atmos. Health.

[9] Nhung N.T.T., Amini H., Schindler C., Kutlar Joss M., Dien T.M., Probst-Hensch N., Perez L., Künzli N. (2017). Short-term association between ambient air pollution and pneumonia in children: A systematic review and meta-analysis of time-series and case-crossover studies. Environ. Pollut..

[10] Zhang Z., Hong Y., Liu N. (2017). Association of ambient Particulate matter 2.5 with intensive care unit admission due to pneumonia: A distributed lag non-linear model. Sci. Rep..

[11] Brauer M., Hoek G., Van Vliet P., Meliefste K., Fischer P.H., Wijga A., Koopman L.P., Neijens H.J., Gerritsen J., Kerkhof M. (2002). Air pollution from traffic and the development of respiratory infections and asthmatic and allergic symptoms in children. Am. J. Respir. Crit. Care Med..

[12] Brunekreef B., Janssen N.A., de Hartog J., Harssema H., Knape M., van Vliet P. (1997). Air pollution from truck traffic and lung function in children living near motorways. Epidemiology.

[13] Brunekreef B., Stewart A.W., Anderson H.R., Lai C.K., Strachan D.P., Pearce N., the ISAAC Phase 3 Study Group (2009). Self-reported truck traffic on the street of residence and symptoms of asthma and allergic disease: A global relationship in ISAAC phase 3. Environ. Health Perspect..

[14] Eckel S.P., Berhane K., Salam M.T., Rappaport E.B., Linn W.S., Bastain T.M., Zhang Y., Lurmann F., Avol E.L., Gilliland F.D. (2011). Residential traffic-related pollution exposures and exhaled nitric oxide in the children’s health study. Environ. Health Perspect..

[15] El-Zein A., Nuwayhid I., El-Fadel M., Mroueh S. (2007). Did a ban on diesel-fuel reduce emergency respiratory admissions for children?. Sci. Total Environ..

[16] Gauderman W.J., Vora H., McConnell R., Berhane K., Gilliland F., Thomas D. (2007). Effect of exposure to traffic on lung development from 10 to 18 years of age: A cohort study. Lancet.

[17] Gonzalez-Barcala F.J., Pertega S., Garnelo L., Castro T.P., Sampedro M., Lastres J.S., Gonzalez M.A., Bamonde L., Valdes L., Carreira J.M. (2013). Truck traffic related air pollution associated with asthma symptoms in young boys: A cross-sectional study. Public Health.

[18] Hu Y., Liu W., Huang C., Zou Z.J., Zhao Z.H., Shen L., Sundell J. (2014). Home dampness, childhood asthma, hay fever, and airway symptoms in Shanghai, China: Associations, dose-response relationships, and lifestyle’s influences. Indoor Air.

[19] Kim J.J., Huen K., Adams S., Smorodinsky S., Hoats A., Malig B., Lipsett M., Ostro B. (2008). Residential Traffic and Children’s Respiratory Health. Environ. Health Perspect..

[20] McConnell R., Islam T., Shankardass K., Jerrett M., Lurmann F., Gilliland F., Gauderman J., Avol E., Künzli N., Yao L. (2010). Childhood incident asthma and traffic-related air pollution at home and school. Environ. Health Perspect..

[21] Wilhelm M., Meng Y.Y., Rull R.P., English P., Balmes J., Ritz B. (2008). Environmental public health tracking of childhood asthma using California health interview survey, traffic, and outdoor air pollution data. Environ. Health Perspect..

[22] Chen F., Lin Z., Chen R., Norback D., Liu C., Kan H., Deng Q., Huang C., Hu Y., Zou Z. (2018). The effects of PM2.5 on asthmatic and allergic diseases or symptoms in preschool children of six Chinese cities, based on China, Children, Homes and Health (CCHH) project. Environ. Pollut..

[23] Gao J., Woodward A., Vardoulakis S., Kovats S., Wilkinson P., Li L., Xu L., Li J., Yang J., Li J. (2017). Haze, public health and mitigation measures in China: A review of the current evidence for further policy response. Sci. Total Environ..

[24] Kan H., Chen R., Tong S. (2012). Ambient air pollution, climate change, and population health in China. Environ. Int..

[25] Khreis H., Kelly C., Tate J., Parslow R., Lucas K., Nieuwenhuijsen M. (2017). Exposure to traffic-related air pollution and risk of development of childhood asthma: A systematic review and meta-analysis. Environ. Int..

[26] Liu W., Huang C., Hu Y., Fu Q., Zou Z., Sun C., Shen L., Wang X., Cai J., Pan J. (2016). Associations of gestational and early life exposures to ambient air pollution with childhood respiratory diseases in Shanghai, China: A retrospective cohort study. Environ. Int..

[27] Lu C., Deng Q., Yu C., Sundell J., Ou C. (2014). Effects of ambient air pollution on the prevalence of pneumonia in children: Implication for national ambient air quality standards in China. Indoor Built. Environ..

[28] Lv C., Wang X., Pang N., Wang L., Wang Y., Xu T., Zhang X., Zhou T., Li W. (2017). The impact of airborne particulate matter on pediatric hospital admissions for pneumonia among children in Jinan, China: A case-crossover study. J. Air Waste Manag. Assoc..

[29] Tao Y., Mi S., Zhou S., Wang S., Xie X. (2014). Air pollution and hospital admissions for respiratory diseases in Lanzhou, China. Environ. Pollut..

[30] Wu Y., Zhang S., Hao J., Liu H., Wu X., Hu J., Walsh M.P., Wallington T.J., Zhang K.M., Stevanovic S. (2017). On-road vehicle emissions and their control in China: A review and outlook. Sci. Total Environ..

[31] Zhao Y., Wang S., Lang L., Huang C., Ma W., Lin H. (2017). Ambient fine and coarse particulate matter pollution and respiratory morbidity in Dongguan, China. Environ. Pollut..

[32] Fan X.J., Yang C., Zhang L., Fan Q., Li T., Bai X., Zhao Z.H., Zhang X., Norback D. (2017). Asthma symptoms among Chinese children: The role of ventilation and PM10 exposure at school and home. Int. J. Tuberc. Lung Dis..

[33] Sundell J., Levin H., Nazaroff W.W., Cain W.S., Fisk W.J., Grimsrud D.T., Gyntelberg F., Li Y., Persily A.K., Pickering A.C. (2011). Ventilation rates and health: Multidisciplinary review of the scientific literature. Indoor Air.

[34] Wang X., Liu W., Hu Y., Zou Z., Shen L., Huang C. (2016). Home environment, lifestyles behaviors, and rhinitis in childhood. Int. J. Hyg. Environ. Health.

[35] Holst G.J., Høst A., Doekes G., Meyer H.W., Madsen A.M., Plesner K.B., Sigsgaard T. (2016). Allergy and respiratory health effects of dampness and dampness-related agents in schools and homes: A cross-sectional study in Danish pupils. Indoor Air.

[36] Zheng X.H., Qian H., Zhao Y.L., Shen H.P., Zhao Z.H., Sun Y.X., Sundell J. (2013). Home risk factors for childhood pneumonia in Nanjing, China. Chin. Sci. Bull..

[37] Liu W., Huang C., Hu Y., Zou Z.J., Sundell J. (2013). Associations between indoor environmental smoke and respiratory symptoms among preschool children in Shanghai, China. Chin. Sci. Bull..

[38] Clougherty J.E. (2010). A growing role for gender analysis in air pollution epidemiology. Environ. Health Perspect..

[39] Dong G.H., Chen T., Liu M.M., Wang D., Ma Y.N., Ren W.H., Lee Y.L., Zhao Y.D., He Q.C. (2011). Gender differences and effect of air pollution on asthma in children with and without allergic predisposition: Northeast Chinese children health study. PLoS ONE.

[40] Spencer-Hwang R., Soret S., Ghamsary M., Rizzo N., Baum M., Juma D., Montgomery B. (2016). Gender differences in respiratory health of school children exposed to rail yard-generated air pollution: The ENRRICH study. J. Environ. Health.

[41] Chang J., Liu W., Hu Y., Zou Z., Shen L., Wang X., Cai J., Sun C., Huang C. (2017). Associations between heavy traffic near residence and childhood health outcomes as modified by bedroom floor level and bedroom ventilation. Build. Environ..

[42] Zhou J., Zhao Q., Zhang X., Chen M. (2017). Preliminary investigation on distribution trend of inhalable particles in indoor air at different floors of a building. J. Environ. Hyg..

[43] Zhang Y.P., Li B.Z., Huang C., Yang X., Qian H., Deng Q.H., Zhao Z.H., Li A.G., Zhao J.N., Zhang X. (2013). Ten cities cross-sectional questionnaire survey of children asthma and other allergies in China. Chin. Sci. Bull..

[44] Asher M.I., Keil U., Anderson H.R., Beasley R., Crane J., Martinez F., Mitchell E.A., Pearce N., Sibbald B., Stewart A.W. (1995). International study of asthma and allergies in childhood (ISAAC): Rationale and methods. Eur. Respir. J..

[45] Bornehag C.G., Sundell J., Sigsgaard T. (2004). Dampness in buildings and health (DBH): Report from an ongoing epidemiological investigation on the association between indoor environmental factors and health effects among children in Sweden. Indoor Air.

[46] Wang L., Qu F., Zhang Y., Weschler L.B., Sundell J. (2015). Home environment in relation to allergic rhinitis among preschool children in Beijing, China: A cross-sectional study. Build. Environ..

[47] Ministry of Construction of the People’s Republic of China (2005). Code for Design of Civil Building (GB50352-2005).

[48] Dales R., Wheeler A., Mahmud M., Frescura A.M., Smith-Doiron M., Nethery E., Liu L. (2008). The influence of living near roadways on spirometry and exhaled nitric oxide in elementary schoolchildren. Environ. Health Perspect..

[49] Hu Z., Zhao Y.N., Cheng Y., Guo C.Y., Wang X., Li N., Liu J.Q., Kang H., Xia G.G., Hu P. (2016). Living near a major road in Beijing: Association with lower ling function, airway acidification, and chronic cough. Chin. Med, J..

[50] Brown M.S., Sarnat S.E., DeMuth K.A., Brown L.A., Whitlock D.R., Brown S.W., Tolbert P.E., Fitzpatrick A.M. (2012). Residential proximity to a major roadway is associated with features of asthma control in children. PLoS ONE.

[51] Perez L., Lurmann F., Wilson J., Pastor M., Brandt S.J., Künzli N., McConnell R. (2012). Near-roadway pollution and childhood asthma: Implications for developing “win-win” compact urban development and clean vehicle strategies. Environ. Health Perspect..

[52] Ryan P.H., LeMasters G., Biagini J., Bernstein D., Grinshpun S.A., Shukla R., Wilson K., Villareal M., Burkle J., Lockey J. (2005). Is it traffic type, volume, or distance? Wheezing in infants living near truck and bus traffic. J. Allergy Clin. Immunol..

[53] Shirinde J., Wichmann J., Voyi K. (2015). Allergic rhinitis, rhinoconjunctivitis and hay fever symptoms among children are associated with frequency of truck traffic near residences: A cross sectional study. Environ. Health.

[54] Jung K.H., Bernabé K., Moors K., Yan B., Chillrud S.N., Whyatt R., Camann D., Kinney P.L., Perera F.P., Miller R.L. (2011). Effects of floor level and building type on residential levels of outdoor and indoor polycyclic aromatic hydrocarbons, black carbon, and particulate matter in New York City. Atmosphere.

[55] Yang F., Kang Y., Gao Y., Zhong K. (2015). Numerical simulations of the effect of outdoor pollutants on indoor air quality of buildings next to a street canyon. Build. Environ..

[56] Zhang Y., Kwok K.C., Liu X.P., Niu J.L. (2015). Characteristics of air pollutant dispersion around a high-rise building. Environ. Pollut..

[57] Zhao Z., Zhang Z., Wang Z., Ferm M., Liang Y., Norbäck D. (2008). Asthmatic symptoms among pupils in relation to winter indoor and outdoor air pollution in schools in Taiyuan, China. Environ. Health Perspect..

[58] Sun Y.X., Sundell J. (2013). On associations between housing characteristics, dampness and asthma and allergies among children in Northeast Texas. Indoor Built. Environ..

[59] Cai J., Liu W., Huang C., Wang X., Shen L., Zou Z., Hu Y., Sun C., Wei X., Chang J. (2016). Validity of subjective questionnaire in evaluating dwelling characteristics, home dampness, and indoor odors in Shanghai, China: Cross-sectional survey and on-site inspection. Energy Build..

[60] Hagerhed-Engman L., Bornehag C.G., Sundell J. (2007). How valid are parents’ questionnaire responses regarding building characteristics, mouldy odour, and signs of moisture problems in Swedish homes?. Scand. J. Public Health.

