# Supplementary material for: Residential Ambient Traffic in Relation to Childhood Pneumonia among Urban Children in Shandong, China: A Cross-Sectional Study
Source: Int J Environ Res Public Health. 2018 May 25;15(6):1076. doi: 10.3390/ijerph15061076 (PMC6025011; doi:10.3390/ijerph15061076)
Supplement: Supplementary file 1 [file ijerph-15-01076-s001.pdf]

Supplemental Materials

Residential Ambient Traffic in Relation to Childhood Pneumonia among Urban Children in Shandong, China: A Cross-Sectional Study

Jing Chang [1,2], Wei Liu [3,4] and Chen Huang [1,*]

[1] School of Environment and Architecture, University of Shanghai for Science and Technology, Shanghai, China; changjingcj2004@163.com
[2] Department of Thermal Energy and Power Engineering, Shandong Jiaotong University, Jinan, China
[3] Department of Building Science, Tsinghua University, Beijing, China; lw1987@tsinghua.edu.cn
[4] Beijing Key Laboratory of Indoor Air Quality Evaluation and Control, Tsinghua University, Beijing, China
* Correspondence: hcyhyywj@163.com or huangc@usst.edu.cn

Commented [M1]: Please carefully check the accuracy of names and affiliations. Changes will not be possible after proofreading.

Commented [M2]: Affil 1–4: Please add post code. (or zip code in the US).

Contents

SI-1. Questionnaire used in CCHH phase I

Table S1. Correlation among family habits of window opening in different seasons.

Table S2. Correlation among residential ambient traffic-related indicators

Table S3. Associations of indictors for residential traffic with childhood pneumonia in the logistic analysis for multiple exposures

Table S4. Dose-response relationships of the cumulative number of indictors for residential traffic with odds of childhood pneumonia

Table S5. Dose-response relationships of the cumulative number of indictors for residential traffic with odds of childhood pneumonia, stratified by the child's sex

Table S6. Dose-response relationships of the cumulative number of indictors for residential traffic with odds of childhood pneumonia, stratified by bedroom floor level

Table S7. Dose-response relationships of the cumulative number of indictors for residential traffic with odds of childhood pneumonia, stratified by family habit of bedroom ventilation

SI-1. Questionnaire used in CCHH phase I

ID:________

China Children Homes Health (CCHH) Group

Study on children's health and home environment exposure

China – Children – Homes – Health

Note: Please fill in the blank on the line or choose the answer with" √"in " □".

Basic information

Who filled in the survey?

- □ Father
- □ Mother
- □ Grandmother
- □ Grandfather
- □ Others

What was the weight and height of the child at birth? ____kg; _____cm

What is the current weight and height of the child? ____kg; _____cm

How old was the mother when the child was given birth? _____years

In which month was the child born? (month) _____

Where was the child born? (province)______

When was the survey completed? (year)____(month)_____(date)_____

Background information of the child and the family

1a. In which week of pregnancy was the child born?

- □ Before month 8 (before week 34)
- □ Month 8-8.5 (in week 34 – 36)
- □ Month 8.5-9 (in week 36 – 38)
- □ Month 9-9.5 (in week 38-40)
- □ After month 9 (after week 40)

- ☐ Not known

2a. Gender of the child?

- ☐ Male
- ☐ Female

2b. Age of the child?

- ☐ 1 year
- ☐ 2 years
- ☐ 3 years
- ☐ 4 years
- ☐ 5 years
- ☐ 6 years
- ☐ 7 years
- ☐ 8 years

4. How long has the child been breastfed totally or partly?

- ☐ No breast-feeding
- ☐ < 1 month
- ☐ 1-2 months
- ☐ 3-6 months
- ☐ > 6 months

5. At what age was the child first given infant formula, gruel or porridge?

- ☐ Younger than 3 months
- ☐ 3-6 months
- ☐ Older than 6 months

6. At what age was the child first given tasters (samples) of food, e.g. fruit purées, mashed root vegetables (e.g. potatoes)?

- ☐ Younger than 3 months
- ☐ 3-6 months
- ☐ Older than 6 months

7. What kind of napkins (Am. diapers) is <u>most commonly</u> used?

- ☐ Nappy
- ☐ Disposable nappy
- ☐ Other kind

8. How many children, who are less than 8 years old, are permanently living at home?__________

9. How many persons, in total, are permanently living at home?__________

10. Whose family does the child stay with permanently?

- ☐ Parents
- ☐ Grandparents
- ☐ Both
- ☐ Others

11. Who takes care of the child before the child starts to attend day nursery?

- ☐ Parents
- ☐ Grandparents
- ☐ Nanny or others

12. **If the child has stayed at day nursery, at what age did the child start to attend it?**
    - 2 years of age or younger
    - 3 years of age or older
13. **The occupation of the mother during pregnancy:**
    - Unemployed
    - Farmer
    - Teacher
    - Office worker
    - Housewife
    - Medical work
    - At factory
    - Salesperson
    - Student
    - Other

The child's and the family's health

14. Has your child <u>ever</u> had wheezing or whistling in the chest at any time in the past? (More than one alternative possible)

- Yes, prior to 1 year of age
- Yes, 1 – 2 years of age
- Yes, 3 – 4 years of age
- Yes, more than 4 years of age
- No

15. Has your child had wheezing or whistling in the chest <u>in the last 12 months</u>? (More than one alternative possible)

- Yes, when having a cold
- Yes, during exercise
- Yes, when playing or being outdoors
- Yes, when laughing or crying
- Yes, in contact with furred animals
- No

16. <u>In the last 12 months</u>, has your child had a dry cough at night for more than two weeks, apart from a cough associated with a cold or chest infection?

- Yes
- No

17. Has your child been diagnosed with asthma by a doctor?

- Yes
- No

18. Has your child had croup (breathing difficulties with server (dry) cough)?

- Yes
- No

19. Has your child been diagnosed with pneumonia by a doctor?

- Yes
- No

20. Has your child ever had a problem with sneezing, or a runny, or a blocked nose when he / she did not have a cold or a flu? (More than one alternative possible)

- Yes, prior to 1 year of age
- Yes, 1-2 years of age
- Yes, 3-4 years of age
- Yes, after 4 years of age
- No

21. In the past 12 months, has your child had a problem with sneezing, or a runny, or a blocked nose when he / she did not have a cold or the flu?

- Yes
- No

22. In the past 12 months, has your child had a problem with sneezing, a runny or a blocked nose, or itchy-watery eyes after been in contact with furred animals?

- Yes
- No

23 (a). In the past 12 months, has your child had a problem with sneezing, a runny or a blocked nose, or itchy-watery eyes after been in contact with pollen?

- Yes
- No

23 (b). During the past 12 months, in which months your child had the problem with sneezing, a runny or a blocked nose, or itchy-watery eyes after been in contact with pollen? (More than one alternative possible)

- January
- February
- March
- April
- May
- June
- July
- August
- September
- October
- November
- December

24. Has your child been diagnosed with hay fever or allergic rhinitis by a doctor?

- Yes
- No

25. In the past 12 months, how many times has your child had a cold?

- Less than 3 times
- 3-5 times
- 6-10 times
- More than 10 times

26. How long does usually a cold last?

- Less than 2 weeks
- 2-4 weeks
- More than 4 weeks

27. Has your child ever had inflammations of the ears?

- Yes, 1-2 times
- Yes, 3-5 times
- Yes, more than 5 times
- No

28 (a) Has your child ever had an itchy rash (eczema), which was coming and going for the last 6 months?

- Yes, prior to 1 year of age
- Yes, 1-2 years of age
- Yes, 3- 4 years of age
- Yes, after 4 years of age
- No

28 (b) Has this itchy rash at any time affected any of the following places: the fold of the elbows, behind the knees, in front of the ankles, under the buttocks, or around the neck, ears or eyes?

- Yes
- No

28 (c) Has your child had this itchy rash at any time in the last 12 months?

- Yes
- No

28 (d) In the last 12 months, how often, on average, has your child been kept awake at night by this itchy rash?

- Never
- Less than one night per week
- One or more nights per week

29. Has the child at any time had allergic irritations such as eczema, nettle-rash, diarrhoea, swollen lips or eyes caused by the listed foods below? (More than one alternative possible)

- Yes, egg
- Yes, sea food
- Yes, meat
- Yes, vegetable
- Yes, flour
- Yes, bean
- Yes, fruit
- Yes, milk or dairy product

- Yes, nut, (peanut, walnut etc.)
- Yes, other
- No
- Not known

30. Has the child been taking medicines with antibiotics, e.g. penicillin? (More than one alternative possible)

- Yes, when 0-12 months old
- Yes, when 12-24 months old
- Yes, after 24 months old
- No, never

31. If the child was taking antibiotics when 0-12 months old, how many treatments did he/she receive?

- 1 treatment
- 2 treatments
- 3 or more treatments

32 (a) Do asthma or allergic problems exist in the family?

- Yes
- No

32 b) If Yes, which kind of problems and for whom? (More than one alternative possible)

Biological father

- Asthma
- Allergic nose or eyes problems
- Eczema

Biological mother

- Asthma
- Allergic nose or eyes problems
- Eczema

Full or half siblings

- Asthma
- Allergic nose or eyes problems
- Eczema

Grandparents

- Asthma
- Allergic nose or eyes problems
- Eczema

33. Does any of the following persons in your household cough without having a cold?

Father

- Yes
- No

Mother

- Yes
- No

Grandparents
- Yes
- No

Siblings
- Yes
- No

34. How many times have you had colds in your household the past year?

Grandparents
- None
- 1-2 times
- 3-4 times
- 5 or more times

Father
- None
- 1-2 times
- 3-4 times
- 5 or more times

Mother
- None
- 1-2 times
- 3-4 times
- 5 or more times

Siblings
- None
- 1-2 times
- 3-5 times
- 6-10 times
- 10 or more times

35. Do you feel cold in your home in winter?
- Every day
- Often (every week)
- Sometimes
- Never

36. During the last 3 months, have you (parent or guardian who complete this survey) had any (one or more) of the following symptoms? (More than one alternative possible)

Fatigue
- Yes, often (every week)
- Yes, sometimes
- No, never

Feeling heavy-headed
- Yes, often (every week)
- Yes, sometimes
- No, never

Headache

- Yes, often (every week)
- Yes, sometimes
- No, never

Nausea / dizziness

- Yes, often (every week)
- Yes, sometimes
- No, never

Difficulties concentrating

- Yes, often (every week)
- Yes, sometimes
- No, never

Itching, burning or irritation of the eyes

- Yes, often (every week)
- Yes, sometimes
- No, never

Irritating, stuffy or runny nose

- Yes, often (every week)
- Yes, sometimes
- No, never

Hoarse, dry throat

- Yes, often (every week)
- Yes, sometimes
- No, never

Cough

- Yes, often (every week)
- Yes, sometimes
- No, never

Dry or flushed facial skin

- Yes, often (every week)
- Yes, sometimes
- No, never

Scurfing / itching scalp or ears

- Yes, often (every week)
- Yes, sometimes
- No, never

Hands dry, itching, red skin

- Yes, often (every week)
- Yes, sometimes
- No, never

Joint pain

- Yes, often (every week)
- Yes, sometimes
- No, never

The residence of the child

37. Has the child been living at the present residence during the whole of his/her life?

- Yes
- No, lived here since year________

39 (a). Where is the residence situated?

- Inner city area
- Suburban
- Rural area (countryside)

39 (b). Surrounding environment (within 200 meters) of your residence (More than one alternative possible)

- Highway
- River or lake
- Commercial district
- Industrial district
- Other

39 (c). Window types of your residence?

Glass in frames is:

- Single layer
- Double layers
- Three layers

40. Window frames are:

- Wooden
- Other

41 (a). In which kind of house is the child living in at the moment?

- Single-family dwelling
- Attached or semi-attached dwelling
- Flat / apartment in multi-family dwelling
- Other

41 (b). How many floors of this house? _________

41 (c). Child's room is in which floor? _________

42. Can you approximately estimate the size of your residence?

- Smaller than 40 sq. m. (m^2)
- 41-60 sq. m. (m^2)
- 61-75 sq. m. (m^2)
- 76-100 sq. m. (m^2)
- 101-150 sq. m. (m^2)
- 150 or larger than 150 sq. m. (m^2)

43. Can you state, approximately, the year that the residence was built?

- Prior to 1980
- 1980-1990
- 1991-2000
- 2001-2005
- 2006 -until now

44. Are you the owner of the residence?

- ☐ Yes
- ☐ No

46. In which room does the child sleep most of his/her sleeping time?

- ☐ The child's own room
- ☐ Sharing bed room with siblings (brothers and sisters)
- ☐ Sleeping with grandparents
- ☐ Sleeping with parents

49. State which kind of flooring material there is in the child's room

- ☐ Wooden floor
- ☐ Laminate wooden floor
- ☐ Bamboo
- ☐ Tile and stones
- ☐ Cement
- ☐ PVC(plastic)
- ☐ Chemical fibre carpet
- ☐ Wool carpet
- ☐ Hemp carpet
- ☐ Not known

50. Which kind of surface layer is on the walls in the child's room?

- ☐ Wallpaper
- ☐ Paint
- ☐ Wooden panel
- ☐ Lime
- ☐ Cement
- ☐ Latex paint
- ☐ Other

52. Which type of heating is there in the residence? (More than one alternative possible)

- ☐ Coal stove
- ☐ Kang
- ☐ Fire wall
- ☐ Fire basin
- ☐ Electric heater
- ☐ Air conditioner heating
- ☐ Floor heating
- ☐ District heating
- ☐ Individual heating
- ☐ Other
- ☐ No heating

53. Which fuel do you generally use for cooking? (More than one alternative possible)

- ☐ Coal
- ☐ Wood
- ☐ Gas

- Electricity
- Other

54 (a). What kind of exhaust equipment are there in your kitchen? (More than one alternative possible)

- Smoke exhaust ventilator
- Exhaust fan
- No

54 (b). Is there any exhaust fan in your bathroom?

- Yes
- No

54 (c). Where is the bathroom air exhausted to?

- Outside the residence
- Inside the residence (such as corridor and staircase, etc)

56. Have you bought new furniture during the period of time stated below?

Within one year before pregnancy

- Yes, a few
- Yes, many
- No

During pregnancy

- Yes, a few
- Yes, many
- No

When the child was 0-1 years old

- Yes, a few
- Yes, many
- No

When the child is more than 1 year old

- Yes, a few
- Yes, many
- No

57. Have you renovated your home during the period of time stated below?

Within one year before pregnancy

- Yes
- No
- Not known

During pregnancy

- Yes
- No
- Not known

When the child was 0-1 years old

- Yes
- No
- Not known

When the child was more than 1 year old

- Yes
- No
- Not known

58. Have you noticed any <u>visible mould</u> on the floor, walls or ceiling in the child's room?

- Yes
- No
- Not known

59. Have you noticed any <u>visible damp stains</u> on the floor, walls or ceiling in the child's room?

- Yes
- No
- Not known

60. How often is the window open when the child is sleeping during the night?

Spring:

- Never
- Sometimes
- Often

Summer:

- Never
- Sometimes
- Often

Autumn:

- Never
- Sometimes
- Often

Winter:

- Never
- Sometimes
- Often

63 (a). Have you noticed your clothing and/or bedding are damp in the last year?

- Never
- Sometimes
- Often

63b. Have your bedding been sun-cured?

- Never
- Sometimes
- Often

64. Has there been any flooding or other kind of water damage in your residence?

- Yes, in the past years
- Yes, in the last 12 months
- No
- Not known

66. In the winter, does condensation or moisture occur on the inside, at the bottom, of windows in the child's room?

- ☐ No, never
- ☐ Yes, less than 5 centimetres
- ☐ Yes, 5-25 centimetres
- ☐ Yes, more than 25 centimetres
- ☐ Not known

67. Have you during the last 3 months been bothered by any (one or more) of the odours, stated below, in your residence?

Stuffy "bad" smell

- ☐ Yes, frequently (weekly)
- ☐ Yes, sometimes
- ☐ No, never

Unpleasant smell

- ☐ Yes, frequently (weekly)
- ☐ Yes, sometimes
- ☐ No, never

Pungent smell

- ☐ Yes, frequently (weekly)
- ☐ Yes, sometimes
- ☐ No, never

Mouldy smell

- ☐ Yes, frequently (weekly)
- ☐ Yes, sometimes
- ☐ No, never

Tobacco smoke

- ☐ Yes, frequently (weekly)
- ☐ Yes, sometimes
- ☐ No, never

Air humid

- ☐ Yes, frequently (weekly)
- ☐ Yes, sometimes
- ☐ No, never

Air dry

- ☐ Yes, frequently (weekly)
- ☐ Yes, sometimes
- ☐ No, never

68a. Have you noticed any animals, stated below, in your residence?

Cockroach

- ☐ Always
- ☐ Often
- ☐ Sometimes
- ☐ No

Mice

- Always
- Often
- Sometimes
- No

Mosquitoes and flies

- Always
- Often
- Sometimes
- No

68(b). Have you used mosquito coils or mosquito repellents in your residence?

- Yes, often
- Yes, sometimes
- No

68(c). Have you used incense in your residence?

- Yes, often
- Yes, sometimes
- No

69 (a). Did you notice there are visible mould or damp stains on the floor, walls or ceiling in the child's residence at birth?

- Yes, often (weekly)
- Yes, sometimes
- No, never

69 (b). Did you notice there are condensation or moisture occur on <u>the inside,</u> at the bottom, of windows in winter in the child's residence at birth?

- Yes, often (weekly)
- Yes, sometimes
- No, never

70. Have you been bothered by any (one or more) of the odours, stated below, in the child's residence at birth?

Stuffy "bad" smell

- Often
- Sometimes
- No

Unpleasant smell

- Often
- Sometimes
- No

Pungent smell

- Often
- Sometimes
- No

Mouldy smell

- Often
- Sometimes
- No

Tobacco smoke

- Often
- Sometimes
- No

Dry air

- Often
- Sometimes
- No

Damp air

- Often
- Sometimes
- No

Questions concerning habits and customs

71. Do you have any furred animals / pets in your present residence?

- Yes, cats
- Yes, dogs
- Yes, rodents (rabbits, rats, etc.)
- Yes, birds
- Yes, fish or reptiles
- Yes, the other
- No.

72 Were there any furred animals / pets in the child's residence at birth?

- Yes, cats
- Yes, dogs
- Yes, rodents (rabbits, rats, etc.)
- Yes, birds
- Yes, fish or reptiles
- Yes, the other
- No.

73. Have you got rid of any furred animals / pets due to allergic illnesses in the family?

- Yes
- No

74. Have you refrained from procuring any furred animals / pets due to allergic illnesses in the family?

- Yes
- No

76. How long does your child stay in traffic vehicles per day?

- Less than 30 minutes
- More than 30 minutes

77. How often do you vacuum clean, sweep or mop (wet) the floor in the child's room?

- Every day
- Approx. twice a week
- Once a week
- Every second week
- Once a month
- Less than once a month

78. Have your cleaning routines changed due to allergies in the family?

- Yes
- No
- Not known

79 (a). Do you have (one or more) of the equipment, stated below, in your residence?

More than one alternative possible

- Printer or photocopy machine
- Air humidifier
- Ionizer
- Air conditioner
- Air cleaner

79 (b). Is there a TV set in the child's room?

- Yes
- No

79 (c). Is there a computer in the child's room?

- Yes
- No

80. Does anyone in your family smoke?

- Yes, mother
- Yes, father
- Yes, grandmother
- Yes, grandfather
- Yes, siblings
- Yes, other person
- No

81. Did any of the parents smoke during the child's first year of life?

- Mother
- Father
- No one

82. Did any of the parents smoke during the pregnancy?

- Mother
- Father
- No one

Questions concerning food habits

83 (a). How often does the child eat hamburgers or fried chicken (McDonalds and KFC, etc.) every month?

- 0 time
- 1 time
- 2 times
- 3 times
- 4-10 times
- 11 times or more

83 (b). Please state the food that the child eats often in the following list.

- Instant noodle
- Bread
- Cookies like moon cake or housewife cake, etc.
- Biscuits
- Chips
- French fries
- Fried chicken
- Hamburger
- Popcorn
- Chocolate
- Soft drinks (carbonated drinks or soda water)
- Juice
- Pepper and other spices
- Pickled vegetables
- Instant coffee
- Tea
- Candy
- Jam
- Smoking meat
- Ice cream
- Frozen food
- Canned foods
- Animal pluck

Questions concerning residential ambient traffic

q1. Is the residence close to a main traffic road?

- Yes
- No

q1(a). If q1 is yes, how far is the linear distance (m) between the residence and main traffic road? _________m
q1(b). If q1 is yes, how many lanes are there in the main traffic road near the residence?
_________lane
q1(c). If q1 is yes, do heavy trucks pass along the main traffic road near the residence?

- Yes
- No

q1(d). If q1 is yes, does the bedroom face the main road near the residence?

- Yes
- No

q2. Is the residence close to a filling station?

- Yes
- No

q2(a). If q2 is yes, how far is the linear distance (m) between the residence and the filling station?
_________m
q3. Is the residence close to an automobile 4S shop?

- Yes
- No

q3(a). If q3 is yes, how far is the linear distance (m) between the residence and the automobile 4S shop?_________m
q4. Dies a ground car park exist in the residential community?

- Yes
- No

q4(a). If q4 is yes, how many cars are parked in the ground car park per day on average?_______ cars

Thank you for your participation!

To further investigate the influence of your indoor environment on child's health, we would like to inspect your residence and do medical exam for your child.

Would you like to take the home inspection?

- Yes
- No

Would you like to take your child to the hospital for free medical examination?

- Yes
- No

If "yes", please inform us your contact information:
Mobile phone: ___________________

Home address: ___________________

Home phone: ____________________

Table S1. Correlation among family habits of window opening in different seasons

	Pearson's correlation coefficient [a]			
	**Spring**	**Summer**	**Autumn**	**Winter**
Spring	1.000	0.477	0.726	0.553
Summer		1.000	0.520	0.260
Autumn			1.000	0.580
Winter				1.000

[a] All p-values were <0.001.

Table S2. Correlation among residential ambient traffic-related indicators

	Pearson's correlation coefficient [a]			
	**Main traffic road**	**Filling station**	**Automobile 4S shop**	**Ground car park**
Main traffic road	1.000	0.166	0.106	0.069
Filling station		1.000	0.252	0.046
Automobile 4S shop			1.000	0.055
Ground car park				1.000

[a] All p-values were <0.001.

Table S3. Associations of indictors for residential traffic with childhood pneumonia in the logistic analysis for multiple exposures

**Indictors for residential traffic (yes *vs.* no)**	**OR, 95%CI (*p*-value)** [a]		
	Crude	Adjusted model 1 [b]	Adjusted model 2 [c]
Residences close to main traffic road near within 200 m	**1.24, 1.09-1.42 (<0.001)**	**1.18, 1.03-1.36 (0.018)**	**1.17, 1.02-1.35 (0.030)**
Residences close to filling station within 200 m	1.09, 0.80-1.48 (0.587)	1.06, 0.76-1.47 (0.739)	1.07, 0.76-1.50 (0.692)
Rsidences close to automobile 4S shop within 200 m	1.31, 0.86-1.99 (0.205)	1.50, 0.95-2.35 (0.081)	1.24, 0.78-1.97 (0.356)
Residential communities having ground car parks	**1.40, 1.23-1.60 (<0.001)**	**1.21, 1.05-1.40 (0.007)**	**1.18, 1.02-1.37 (0.025)**

[a] OR: odds ratio; CI: confidence interval; Bold indicates significance (p-value<0.05). [b] Multivariate logistic regression analyses with adjustment for the child's sex, age, residence-located area, family history of atopy, residence ownership, breastfeeding duration, household dampness-related exposures, household ETS, and household renovation during early lifetime. [c] Two-level (kindergarten-child) logistic regression analyses with adjustment for the child's sex, age, family history of atopy, residence ownership, breastfeeding duration, household dampness-related exposures, household ETS, and household renovation during early lifetime.

Table S4. Dose-response relationships of the cumulative number of indictors for residential traffic with odds of childhood pneumonia

The cumulative number of indictors for residential traffic	Sample sizes, n (%)	Prevalence, n (%) [*p*-value[a]]	OR, 95%CI (*p*-value)		
			Crude	Adjusted model 1[b]	Adjusted model 2[c]
0	1306 (25.3)	**265 (20.7)**	1.00	1.00	1.00
1	2412 (46.7)	**607 (25.7)**	**1.32, 1.12-1.56 (0.001)**	1.13, 0.95-1.34 (0.185)	1.08, 0.90-1.29 (0.411)
2	1282 (24.8)	**382 (30.4)**	**1.67, 1.39-2.00 (<0.001)**	**1.38, 1.13-1.67 (0.001)**	**1.37, 1.06-1.60 (0.011)**
3	169 (3.3)	**61 (36.5) [<0.001]**	**2.20, 1.56-3.10 (<0.001)**	**1.79, 1.23-2.59 (0.002)**	**1.66, 1.12-2.46 (0.011)**

[a] In the Pearson's chi-square test; Bold indicates significance (*p*-value <0.05). [b] Multivariate logistic regression analyses with adjustment for the child's sex, age, residence-located area, family history of atopy, residence ownership, breastfeeding duration, household dampness-related exposures, household ETS, and household renovation during early lifetime. [c] Two-level (kindergarten-child) logistic regression analyses with adjustment for the child's sex, age, family history of atopy, residence ownership, breastfeeding duration, household dampness-related exposures, household ETS, and household renovation during early lifetime.

Table S5. Dose-response relationships of the cumulative number of indictors for residential traffic with odds of childhood pneumonia, stratified by the child's sex

The cumulative number of indictors for residential traffic	Sample sizes, n (%)	Prevalence, n (%) [*p*-value[a]]	OR, 95%CI (*p*-value)		
			Crude	Adjusted model 1[b]	Adjusted model 2[c]
Boys					
0	632 (23.7)	**139 (22.5)**	1.00	1.00	1.00
1	1245 (46.7)	**326 (26.8)**	**1.26, 1.01-1.58 (0.047)**	1.10, 0.86-1.40 (0.436)	1.06, 0.83-1.37 (0.634)
2	693 (26.0)	**203 (29.7)**	**1.46, 1.14-1.87 (0.003)**	1.23, 0.94-1.61 (0.136)	1.22, 0.91-1.62 (0.179)
3	95 (3.6)	**37 (39.8) [0.001]**	**2.28, 1.44-3.59 (<0.001)**	**2.20, 1.36-3.57 (0.001)**	**2.16, 1.28-3.65 (0.004)**
Girls					
0	647 (26.6)	**123 (19.3)**	1.00	1.00	1.00
1	1138 (46.8)	**275 (24.6)**	**1.36, 1.07-1.73 (0.011)**	1.16, 0.90-1.49 (0.257)	1.13, 0.88-1.47 (0.337)
2	577 (23.7)	**176 (31.3)**	**1.90, 1.46-2.47 (<0.001)**	**1.56, 1.17-2.07 (0.002)**	**1.42, 1.05-1.91 (0.023)**
3	71 (2.9)	**22 (31.0) [<0.001]**	**1.87, 1.09-3.21 (0.021)**	1.30, 0.71-2.37 (0.392)	1.18, 0.62-2.22 (0.614)
p-value for interaction			**<0.001**	**0.002**	**0.006**

[a] In the Pearson's chi-square test; Bold indicates significance (p-value <0.05). [b] Multivariate logistic regression analyses with adjustment for the child's age, residence-located area, family history of atopy, residence ownership, breastfeeding duration, household dampness-related exposures, household ETS, and household renovation during early lifetime. [c] Two-level (kindergarten-child) logistic regression analyses with adjustment for the child's age, family history of atopy, residence ownership, breastfeeding duration, household dampness-related exposures, household ETS, and household renovation during early lifetime.

Table S6. Dose-response relationships of the cumulative number of indictors for residential traffic with odds of childhood pneumonia, stratified by bedroom floor level

**The cumulative number of indictors for residential traffic**	**Sample sizes, n (%)**	**Prevalence, n (%) [p-value[a]]**	**OR, 95%CI (p-value)**		
			**Crude**	**Adjusted model 1[b]**	**Adjusted model 2[c]**
1-3 floors					
0	745 (29.8)	**147 (20.1)**	1.00	1.00	1.00
1	1139 (45.5)	**295 (26.6)**	**1.44, 1.15-1.80 (0.001)**	**1.30, 1.02-1.65 (0.035)**	1.20, 0.94-1.55 (0.149)
2	541 (21.6)	**165 (31.2)**	**1.81, 1.40-2.35 (<0.001)**	**1.58, 1.19-2.09 (0.001)**	**1.51, 1.13-2.02 (0.006)**
3	78 (3.1)	**24 (31.6) [<0.001]**	**1.84, 1.10-3.08 (0.019)**	1.39, 0.78-2.47 (0.266)	1.23, 0.67-2.27 (0.499)
4-6 floors					
0	369 (19.5)	**90 (24.9)**	1.00	1.00	1.00
1	938 (49.6)	**216 (23.4)**	0.92, 0.69-1.22 (0.557)	0.78, 0.58-1.06 (0.111)	0.80, 0.59-1.09 (0.162)
2	520 (27.5)	**156 (30.5)**	1.32, 0.97-1.79 (0.073)	1.08, 0.77-1.50 (0.663)	1.11, 0.78-1.57 (0.574)
3	63 (3.4)	**26 (41.3) [0.001]**	**2.12, 1.21-3.69 (0.007)**	**1.86, 1.01-3.41 (0.046)**	1.85, 0.97-3.54 (0.063)
≥7 floors					
0	88 (16.4)	19 (21.8)	1.00	1.00	1.00
1	236 (44.1)	73 (31.5)	1.64, 0.92-2.93 (0.091)	1.17, 0.62-2.20 (0.621)	1.15, 0.61-2.15 (0.662)
2	185 (34.6)	52 (28.6)	1.43, 0.78-2.61 (0.241)	1.66, 0.80-3.42 (0.172)	1.63, 0.78-2.44 (0.193)
3	26 (4.9)	9 (34.6) [0.353]	1.90, 0.73-4.92 (0.185)	2.69, 0.78-9.27 (0.118)	2.29, 0.72-7.32 (0.163)
p-value for interaction			**0.004**	0.082	0.108

[a] In the Pearson's chi-square test; Bold indicates significance (p-value <0.05). [b] Multivariate logistic regression analyses with adjustment for the child's sex, age, residence-located area, family history of atopy, residence ownership, breastfeeding duration, household dampness-related exposures, household ETS, and household renovation during early lifetime. [c] Two-level (kindergarten-child) logistic regression analyses with adjustment for the child's sex, age, family history of atopy, residence ownership, breastfeeding duration, household dampness-related exposures, household ETS, and household renovation during early lifetime.

Table S7. Dose-response relationships of the cumulative number of indictors for residential traffic with odds of childhood pneumonia, stratified by family habit of bedroom ventilation

**The cumulative number of indictors for residential traffic**	**Sample sizes, n (%)**	**Prevalence, n (%) [*p*-value[a]]**	**OR, 95%CI (*p*-value)**		
			**Crude**	**Adjusted model 1[b]**	**Adjusted model 2[c]**
Often open the bedroom windows during night					
0	903 (24.7)	**187 (21.1)**	1.00	1.00	1.00
1	1717 (47.0)	**426 (25.3)**	**1.27, 1.04-1.54 (0.018)**	1.07, 0.87-1.32 (0.506)	1.05, 0.84-1.30 (0.686)
2	918 (25.2)	**277 (30.7)**	**1.66, 1.34-2.06 (<0.001)**	**1.41, 1.11-1.77 (0.004)**	**1.34, 1.05-1.71 (0.018)**
3	112 (3.1)	**34 (30.6) [<0.001]**	**1.65, 1.07-2.55 (0.023)**	1.37, 0.86-2.19 (0.188)	1.32, 0.81-2.16 (0.258)
Not often open the bedroom windows during night					
0	362 (25.4)	**73 (20.7)**	1.00	1.00	1.00
1	658 (46.2)	**176 (27.3)**	**1.43, 1.05-1.96 (0.022)**	1.12, 0.76-1.65 (0.581)	1.09, 0.72-1.64 (0.687)
2	350 (24.6)	**98 (28.6)**	**1.53, 1.08-2.17 (0.017)**	1.24, 0.89-1.72 (0.209)	1.24, 0.89-1.74 (0.203)
3	54 (3.8)	**26 (49.1) [<0.001]**	**3.68, 2.03-6.69 (<0.001)**	**2.83, 1.44-5.59 (0.003)**	**2.89, 1.41-5.89 (0.004)**
p-value for interaction			**0.001**	0.065	0.051

[a] In the Pearson's chi-square test; Bold indicates significance (*p*-value <0.05). [b] Multivariate logistic regression analyses with adjustment for the child's sex, age, residence-located area, family history of atopy, residence ownership, breastfeeding duration, household dampness-related exposures, household ETS, and household renovation during early lifetime. [c] Two-level (kindergarten-child) logistic regression analyses with adjustment for the child's sex, age, family history of atopy, residence ownership, breastfeeding duration, household dampness-related exposures, household ETS, and household renovation during early lifetime.
